# Immunotherapy with heat shock protein 96 to treat gliomas

**DOI:** 10.1186/s41016-020-00211-3

**Published:** 2020-09-03

**Authors:** Chunzhao Li, Yifei Du, Yang Zhang, Nan Ji

**Affiliations:** 1 Department of Neurosurgery, Beijing Tiantan Hospital, Capital Medical University, No.119 South 4th Ring West Road, Fengtai District, Beijing, 100070 China; 2 Beijing Shijitan Hospital, Capital Medical University, No.10 Yangfangdian Road, Fengtai District, Beijing, 100038 China; 3 China National Clinical Research Center Neurological Diseases, Beijing Tiantan Hospital, Capital Medical University, No.119 South 4th Ring West Road, Fengtai District, Beijing, 100070 China; 4 Beijing Key Laboratory of Brian Tumor, Beijing Tiantan Hospital Capital Medical University, No.119 South 4th Ring West Road, Fengtai District, Beijing, 100070 China

**Keywords:** Glioma, gp96, Tumor vaccine, Oncogenesis

## Abstract

Heat shock protein 96 (gp96) is a highly conserved protein in the endoplasmic reticulum. The functions of gp96 include promoting the oncogenesis and progression of glioma. In addition, tumor-derived gp96 can activate anti-tumor immune. Therefore, this protein was used to generate an anti-tumor vaccine and widely applied to glioma therapy. This review summarizes the mechanisms of gp96 in glioma oncogenesis and clinical trials of the gp96 tumor vaccine in glioma treatment.

## Background

Heat shock protein glycoprotein 96 kDa (gp96) is also named glucose-regulated protein 94 (GRP94), endoplasmic reticulum protein 99 (ERp99), and heat shock protein 90 b1 (HSP90b1). As with other heat shock proteins, gp96 expression was significantly increased when the cells were stimulated by hyperthermia, hypoxia, infection, or poisoning. However, because gp96 plays a key role in glioma oncogenesis and anti-tumor immune regulation, it became a therapy for glioma.

## Structure and physiological function of gp96

When classified by its protein molecular weight, gp96 is one of five proteins, with HSP90AA1, HSP90AA2, HSP90AB1, and HSP90L, in the HSP90 family with 50% shared homology. Their structures are conserved and primarily consist of four structural domains: an N-terminal domain, a C-terminal domain, a charged linker region, and a middle domain. Gp96 exerts its functions by binding a variety of proteins in each domain [1]. The primary physiological function of gp96 is facilitation of protein folding, quality control in the endoplasmic reticulum (ER), and regulating Ca^2+^ in cells.

### Facilitating protein folding and ER quality control

The endoplasmic reticulum is an organelle where proteins are synthesized, folded, and transported and is the primary site for posttranscriptional protein modification. Gp96 is a resident protein of the ER that facilitates protein folding and assembly in the ER [2]. However, protein folding is actually an inefficient process that results in many unfolded proteins, which can lead to genetic disease, including diabetes, when accumulated over a specific concentration [3]. The overexpression of unfolded protein in the ER can upregulate the expression of gp96. This upregulation leads to the elevated expression of chaperone protein and the signaling protein IGF- 1 [4]. In addition, gp96 interrupts the transport of abnormal proteins from the ER and activates ubiquitin and proteasomes containing Hrd1 and SEL1L to degrade the unfolded protein through the ER-related degradation process, thus maintaining intracellular homeostasis [5].

### Maintaining intracellular Ca^2+^ homeostasis

In addition to proteins processing, the ER is a regulation and storage site for a variety of ions. Ca^2+^ is an intracellular messenger that is important for signaling, neuronal cell development, synaptic transmission, and plasticity, but excessive Ca^2+^ can lead to cell apoptosis [6]. Gp96 is a low-affinity and high-capacity calcium-binding protein. The strongest Ca^2+^ binding site among the 15 binding sites in gp96 is in the charged domain. Although the Ca^2+^-binding ability of gp96 is not as strong as that of calsequestrin, gp96 effectively maintains homeostasis and prevents death in the presence of calcium ion overload. Researchers have found that caspase-3 and calpain activity, which decrease Ca^2+^ release, are inhibited by increased gp96 [6]. So, gp96 is important for maintaining intracellular Ca^2+^ homeostasis.

## Gp96 promotes glioma oncogenesis and chemoradiotherapy resistance

High expression levels of gp96 have been associated with a high degree of malignancy and poor prognosis for people with one of various tumors [7]. Researchers found that, compared to the normal brain tissue, glioma tissue expressed a higher level of gp96, and patients with a higher gp96 expression level had poorer prognoses [8]. This result is closely related to the function of gp96 in chaperoning oncogenesis proteins in glioma to maintain tumor cell homeostasis and promote proliferation [9]. First, gp96 can promote glioma cell proliferation by maintaining the MAPK and PI3K pathways [10]. In addition, gp96 decreases apoptosis in glioma cells by inhibiting the degradation of retinoblastoma protein and mutant P53 protein [9]. The hypoxia-inducible factor (HIF), which is an inducer of the epithelial-to-mesenchymal transition (EMT) program in epithelial cancers, is chaperoned by gp96 and leads to a mesenchymal phenotype shift and increased invasiveness of glioblastoma (GBM) [11]. Moreover, extracellular gp96 is essential for sustaining AKT and EphA2 activation, further promoting lamellipodia formation and concomitant GBM cell motility and invasion [12]. Furthermore, gp96 regulates TGF-β and Treg cells by assembling GARP, FOXP3, and TGF- β [13]. Integrins and TLRs chaperoned by gp96 indirectly promote tumor progression.

Other studies have demonstrated the roles of gp96 in the oncogenesis of gliomas by knocking it down. After gp96 expression was inhibited, a large number of proteins were degraded in glioma cells, leading to tumor cell apoptosis and autophagy, but no significant effect was found on normal astrocytes [14]. Gp96 depletion has been shown to downregulate the Wnt/β-catenin pathway, which inhibit the migration and invasion of GBM cells [8]. In addition, after the inhibition of gp96, morphological changes and recombination were observed in the actin cytoskeleton of GBM cells, which showed greatly reduced invasiveness [10].

The effect of Gp96 on glioma is reflected not only in oncogenesis but also in resistance to chemoradiotherapy. Researchers have reported that the client proteins of gp96 were able to decrease the radiotherapy sensitivity of the tumor. Survivin is one of the client proteins of gp96, and it can enhance tumor cell survival upon radiation by regulating double-strand DNA break repair and tumor cell metabolism [15]. After the depletion of gp96, the Rad51 and BRCA2 proteins were upregulated, which suppressed the recombination of DNA and enhanced the radiation sensitivity of glioma cells [16]. On the other hand, knockdown of gp96 would enhance the therapeutic effect of temozolomide in GBM [17]. But more researches were needed to disclose the mechanisms of gp96-related chemotherapy resistance in glioma. In general, gp96 is important in glioma oncogenesis because of its chaperone function.

## Gp96 with anti-glioma immunotherapy

### The regulation of Gp96 and cellular immunity

In previous studies, the antigenicity of gp96 peptide complexes was identified by its ability to activate specific cytotoxic T lymphocytes (CTLs) derived from virus-infected cells [18]. Further studies have shown that gp96 was released into the extracellular space, where it bound with an antigen peptide that had effective antigenicity, but only in necrotic cells [19]. Gp96 peptide complexes activated immune responses effectively through endocytosis mediated by the surface receptor CD91 on antigen-presenting cells (APCs) [20]. Endogenous and exogenous antigens were presented on antigen-presenting cells by MHC-I and MHC-II molecules, respectively. The APCs were stimulated to release IL-2 and TNF-α, which promoted the maturation of dendritic cells. Thus, adaptive and innate immunity were activated by gp96 peptide complexes [21]. In general, gp96 activates the immune response faster and more efficiently than other heat shock proteins.

In addition to immune activation, gp96 also plays an important role in maintaining immune system homeostasis, primarily in regulating the immunosuppressive function of Treg cells. When gp96 in Tregs was knocked down, mice suffered lethal inflammation caused by an immune imbalance. Researchers found that gp96 maintained the inhibitory function of Treg by chaperoning the FOXP3 protein in Tregs and activating TGF-β on the cell surface [22]. Thus, gp96 plays an auxiliary role in regulating the immune response.

### Gp96 peptide complexes: novel individualized multivalent tumor vaccine

Due to their powerful adhesive ability and because they present antigens to activate anti-tumor immunity, gp96 peptide complexes were derived from solid tumors and used to create an anti-tumor vaccine. Compared with other tumor vaccines, the gp96 version, as a multivalent tumor vaccine, bound to different types of tumor antigens, which enables it to address tumor heterogeneity better than other treatments and to activate the immune response [23]. In addition, an individual-derived gp96 tumor vaccine had fewer side effects because it was extracted from the patients’ own tumors. In 1984, Srivastava and Das et al. found that the subcutaneous injection of 90 to 100 kDa proteins derived from autologous tumor cells activated humoral immune responses in a liver cancer mouse model, which initiated the use of gp96 in immunotherapy methods [24]. Derived from an autologous tumor and then purified and sterilized, a gp96 tumor vaccine was injected subcutaneously into patients. Early clinical trials on pancreatic adenocarcinoma, melanoma [7], and glioblastoma [23], among others have recently been completed. Because of the special immune microenvironment of the human brain, gp96 immunotherapy for intracranial malignancy was developed relatively late, and information on 7 clinical trials of glioblastoma treatment can be accessed at https://clinicaltrials.gov/ (Table 1). Next, we summarize the results of the different clinical trials to determine the therapeutic effects and mechanism of the gp96 tumor vaccine against glioma.

**Table 1:** Clinical trials of gp96 tumor vaccine in glioma

NCT Number	Start Date	Location	Sponsor/Collaborators	Enrollment	Disease	Interventions	Status
NCT00293423	2005/10/1	US	UCSF Department of Neurosurgery San Francisco, California, United States,	41	Adult glioblastoma;Recurrent adult brain tumor	HSPPC-96^a^ with Temozolomide and radiotherapy	Completed
NCT00905060	2009/6/1	US	University of California, San Francisco	70	Newly diagnosed GBM	HSPPC-96 with Temozolomide and radiotherapy	Completed
NCT01814813	2013/5/1	US	Alliance for Clinical Trials in Oncology	90	Recurrent Glioblastoma;	HSPPC-96 with bevacizumab	Active, not recruiting
T02122822	2013/7/1	China	Beijing Tiantan Hospital Affiliated to Capital Medical University, Beijing, China	20	Supratentoria Glioma	HSPPC-96 with Temozolomide and radiotherapy	Completed
NCT02722512	2016/7/1	US	Ann & Robert H Lurie Children's Hospital of Chicago | Northwestern University	20	GBM; Astrocytoma;Anaplastic Ependymoma	HSPPC-96 with Radiation	Recruiting
NCT03018288	2017/9/21	US	National Cancer Institute (NCI) | National Institutes of Health Clinical Center (CC)	108	Glioblastoma	HSPPC-96 with radiation, temozolomide and Pembrolizumab-96	Recruiting
NCT03650257	2018/8/20	China	Beijing Tiantan Hospital Affiliated to Capital Medical University, Beijing, China	150	Glioma of Brain	HSPPC-96 with Temozolomide and radiotherapy	Recruiting

^a^*HSPPC-96* Heat shock protein peptide complex-96

An early clinical trial on recurrent GBM enrolled 12 patients (NCT00293423) [25] and showed that the gp96 tumor vaccine led only to mild injection site erythema and/or induration. The peripheral blood mononuclear cells (PBMCs) that were extracted preoperatively and postvaccination were stimulated by antigen-binding gp96 (agp96) and recombinant gp96 (without antigen, rgp96), respectively, to test whether antigen binding was necessary for gp96 tumor vaccine therapy. As expected, there was no significant change in the amount of IFN-γ released by PBMCs stimulated by rgp96. However, the PBMCs stimulated by agp96 released 14.1-fold more IFN-γ than the control group. Even the low-response group participants released 3.28-fold more IFN-γ than the control group. Immune responders whose peripheral blood T cells and NK cells increased significantly had a median survival of 47 weeks after surgery and vaccination, compared with 16 weeks for the single nonresponder whose peripheral blood Treg cells had increased. These results showed that gp96 failed to activate the anti-tumor immune response itself, but that gp96 tumor vaccine was able to activate the peripheral and tumor local immune responses; however, for some of the patients, the effect was useless.

In the subsequent phase II clinical trial (NCT00293423), 41 patients with recurrent glioma were recruited [26]. Slight adverse events from the gp96 tumor vaccine were also observed in this study, and the median survival of all the patients was 42.6 weeks. The pre-vaccine absolute lymphocyte count (ALC) from the peripheral blood was tested in the study, and patients with ALC levels below the cohort median demonstrated decreased overall survival (low to high = 49.1 weeks:37.1 weeks). Furthermore, the prevaccination ALC was found to be an independent predictor of the outcome, indicating that patients with better immune function responded better to the gp96 tumor vaccine. In addition, the study found that patients who received the gp96 tumor vaccine multiple times had better outcomes. Because the gp96 vaccine was completely derived from an autologous tumor and the median number of injection times in this study was 6, we can conclude that patients with low tumor load or a low level of gp96 expression may not be suitable recipients of the gp96 tumor vaccine.

Regarding newly diagnosed GBM patients, we finished a phase I clinical trial (NCT02122822) [27] that had enrolled 20 patients. The median survival was 31.4 months, and the median progression-free survival was 6 months, which was longer than that of patients who did not receive the gp96 tumor vaccine. The tumor-specific immune response (TSIR) was tested by IFN-γ release enzyme-linked immunospot (ELISPOT) assay, and the patients were allocated to two groups according their TSIR level. The results showed that the patients with high TSIR had longer overall survival than the low TSIR patients (low to high = 14.6 months:40.5 months). Another phase II clinical trial (NCT00905060) enrolled 46 patients with newly diagnosed GBM [28], and the median overall survival was 23.8 months. The most attractive result in this study was that the programmed death-ligand 1 (PD-L1) expression in myeloid cells was found to be an independent predictor of survival, because a high expression level of PD-L1 in myeloid cells led to shorter survival times (low to high = 18 months:44.7 months).

To summarize these clinical trials, the gp96 tumor vaccine effectively improved the peripheral and local antitumor immune response to prolong overall survival. PBMCs or IFN-γ levels were used in these clinical trials as a reflection of the intensity of the antitumor response, and the results were significantly correlated with prognosis; thus, PBMCs and IFN-γ were effective indicators for the therapeutic effects of the gp96 tumor vaccine. However, the prognosis of patients treated with the gp96 tumor vaccine varied significantly, and some GBM patients did not benefit from in these clinical trials [23]. Orin Bloch found that PD-L1 was a key molecular factor because high PD-L1 expression in PBMCs was associated with the reduced therapeutic effect of the gp96 tumor vaccine [28]. This conclusion suggested that the resistance of the gp96 tumor vaccine may be mediated by an immune checkpoint such as PD-L1. In fact, glioma is a typical “cold” tumor that is infiltrated by a low level of lymphocytes. However, the gp96 tumor vaccine may improve the immune environment by activating peripheral and local anti-tumor immunity. Therefore, it has a synergistic effect with immune checkpoint inhibition therapy, such as anti-PD1. Clinical trials have recently been conducted to explore the therapeutic effect of the gp96 tumor vaccine combined with anti-PD1 (NCT03018288) (ChiCTR2000028998). More relevant studies are needed to clarify the molecular mechanism of combination therapy. Furthermore, finding predictive markers of the therapeutic effects needed to identify suitable patients and then perform precision therapy is another way to improve the efficacy of the vaccine. In addition to the prevaccination expression levels of ALC, PBMCs, and PD-L1 being predictive markers of the gp96 tumor vaccine, we also found that the T cell receptor repertoire was a prognostic marker for the gp96 vaccine [29]. In general, the gp96 tumor vaccine is a useful therapy in glioma but also requires improvement.

Specifically, clinical trials have revealed defects in the gp96 tumor vaccine. The production rate has become the primary problem, as the purification requires many steps. Therefore, some patients cannot receive a sufficient level of the vaccine. In addition, VITESPEN, which has come onto the market as a renal cancer therapy, is still not in widespread use because the advantages are not obvious. Finally, TGF-β, IL-10, and other inhibitory cytokines secreted by the tumor microenvironment may limit the effect of gp96 tumor vaccines, similar to the limits of other immunotherapies [30].

## Conclusion

Gp96 is an essential protein, not only in normal physiological processes but also in the oncogenesis of glioma. The use of a gp96 peptide complex as an individual multivalent tumor vaccine has been shown to be a safe, effective, and promising adjuvant immunotherapy in clinical trials; however, larger and higher quality clinical trials are still needed. In addition, the common defect exposed by all the clinical trials was the significant differences in the therapeutic effect of the gp96 tumor vaccine for individuals. Therefore, relevant research about clarifying the mechanism and finding a predictive marker for the gp96 tumor vaccine must be performed to improve the holistic outcome of the gp96 tumor vaccine as a glioma treatment.

## Data Availability

Not applicable

## Abbreviations

Gp96: Heat shock protein glycoprotein 96 kDa
GBM: Glioblastoma
PD-1: Programmed death 1
PD-L1: Programmed death ligand 1
PBMCs: Peripheral blood mononuclear cells
ALCs: Absolute lymphocyte count
TSIR: Tumor-specific immune response
APCs: Antigen-presenting cells
